# Association between red cell distribution width to albumin ratio and all-cause mortality in stroke survivors: An observational study

**DOI:** 10.1097/MD.0000000000047040

**Published:** 2026-01-09

**Authors:** Lin Zhang, Zhe Yang, Shaoru Xing, Yujia Huo, Yu Wang, Hongxia Du

**Affiliations:** a School of Nursing, Shandong Second Medical University, Weifang, China; b The Second School of Clinical Medicine, Southern Medical University, Guangzhou, China; c School of Nursing, Shandong First Medical University, Jinan, China; d Nursing Department, Central Hospital Affiliated to Shandong First Medical University, Jinan, China.

**Keywords:** mortality, NHANES, RAR, stroke

## Abstract

Stroke is a global cerebrovascular disease. This study mainly explores the association between red cell distribution width to albumin ratio (RAR) and all-cause mortality in stroke survivors, which is helpful for the prognostic management of stroke survivors. Using data from the 1999 to 2018 National Health and Nutrition Examination Survey, Cox regression, restricted cubic spline analysis, receiver operating characteristic curve, and subgroup analysis were applied to assess the relationship between RAR and all-cause mortality in stroke survivors. Sensitivity analysis was also conducted to ensure the robustness of the findings. A total of 1838 stroke survivors were included, with 861 deaths recorded over a median follow-up of 6.42 years. A nonlinear relationship was observed between RAR and all-cause mortality. When RAR was <4.24, it was significantly positively associated with all-cause mortality (hazard ratio = 2.16, 95% confidence interval: 1.77–2.64). In the fully adjusted multivariable model, stroke survivors in the highest quartile of RAR (Q4) had a 1.95 times higher risk of all-cause mortality compared to those in the lowest quartile (Q1). Receiver operating characteristic analysis demonstrated that RAR had good predictive value for all-cause mortality (area under the curve > 0.6). Subgroup analysis showed that there were significant interaction effects between RAR and all-cause mortality in stroke survivors across gender, race, and educational level. Elevated RAR is closely associated with increased all-cause mortality in stroke survivors. This marker may serve as a reliable prognostic indicator for stroke survivors.

## 1. Introduction

Stroke is a critical cerebrovascular condition marked by high disability, elevated mortality, and significant economic costs, presenting a major challenge to global health systems.^[1,2]^ Recent projections indicate that by 2050, global stroke-related deaths will reach 9.7 million, disability-adjusted life years will increase to 189.3 million, and economic losses may amount to up to $2.3 trillion.^[3]^ Against the backdrop of global aging, the burden of stroke continues to escalate. Therefore, exploring novel biomarkers and refining stroke prognosis monitoring and assessment systems are of paramount importance for early identification, prevention, and intervention.

The occurrence and progression of stroke are driven by multiple pathological mechanisms, including atherosclerosis,^[4]^ endothelial dysfunction,^[5]^ inflammatory response,^[6]^ oxidative stress,^[7]^ and thrombosis.^[8]^ Among these, the inflammatory response is present as early as the initial stage of stroke and persists throughout the entire process of brain injury.^[9]^ Following the onset of brain injury, the inflammatory response first activates microglia in the brain, recruits peripheral inflammatory cells into the brain parenchyma, and triggers the release of a large number of proinflammatory cytokines, reactive oxygen species, and matrix metalloproteinases.^[10,11]^ Ultimately, this exacerbates neuronal and brain parenchymal damage, disrupts the blood–brain barrier, and exerts a significant impact on stroke progression.^[10]^ Therefore, focusing on the inflammatory mechanisms after stroke holds guiding significance for the treatment of stroke and the evaluation of its prognosis.

Red cell distribution width (RDW) is a hematological parameter that indicates the variability in red blood cell volume, and an elevated RDW level reflects the body’s inflammatory state.^[12]^ Previous studies have demonstrated the correlation of RDW with predicting stroke-related mortality and poor clinical outcomes.^[13–15]^ During the inflammatory cascade after stroke, cytokines such as interleukin-6 and tumor necrosis factor-α are released. On one hand, these cytokines promote oxidative stress, which causes oxidative damage to the red blood cell membrane and leads to heterogeneous red blood cell maturation.^[13]^ On the other hand, inflammatory cytokines can inhibit bone marrow hematopoietic function and delay erythropoietin-induced red blood cell maturation.^[16]^ Ultimately, these factors collectively result in an increase in RDW levels. Albumin (ALB), the primary component of total serum protein, is widely used to assess systemic inflammatory response and nutritional status, holding significant clinical value in the prognostic evaluation of various diseases.^[17,18]^ The red cell distribution width to albumin ratio (RAR) is a novel potential biomarker. Compared with biomarkers commonly used in current stroke prognosis assessment, such as neuron-specific enolase,^[19]^ C-reactive protein,^[20]^ and D-dimer,^[21]^ the RAR more comprehensively reflects information on inflammatory and nutritional status. Additionally, as a routine indicator, it possesses the advantages of being cost-effective and easily accessible. Currently, RAR has demonstrated significant predictive value in the prognosis assessment of diseases including chronic obstructive pulmonary disease,^[22]^ coronary heart disease,^[23]^ and cancer.^[24]^ Therefore, this research seeks to explore the association between the RAR and all-cause mortality in stroke survivors, utilizing nationally representative data from the National Health and Nutrition Examination Survey (NHANES) database collected from 1999 to 2018 among United States residents. It is anticipated to offer a new biomarker for assessing stroke prognosis risk and provide further evidence to improve stroke prognosis monitoring and evaluation systems.

## 2. Materials and methods

### 2.1. Data availability

The NHANES is a cross-sectional study aimed at gathering comprehensive health and nutrition data from a nationally representative United States population sample, providing a robust foundation for scientific epidemiological analyses. The survey utilizes a multistage sampling methodology to ensure comprehensive representation of national health data characteristics across dimensions such as socioeconomic status, lifestyle behaviors, and disease distribution. Data collection integrates multiple approaches, including questionnaires, physical examinations, household interviews, and laboratory assessments, to thoroughly capture demographic characteristics, nutrient intake, and health risk factors.^[25]^ The data are updated biennially and publicly shared, facilitating diverse research and analytical purposes.

This research, involving human participants, biological specimens, or data, adhered to the Declaration of Helsinki and received approval from the National Center for Health Statistics Ethics Review Board. All individuals provided written consent. For further details or data access, visit the Centers for Disease Control and Prevention website at https://wwwn.cdc.gov/nchs/nhanes/Default.aspx.

### 2.2. Study population

**Figure 1.**
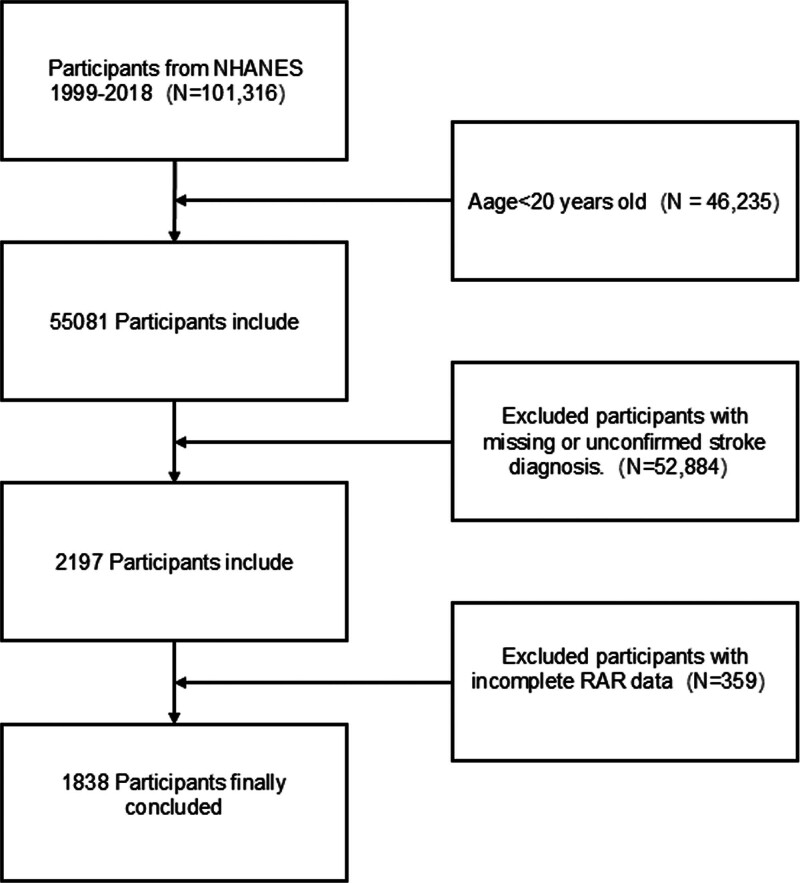
Flow diagram of final sample selection.

In this study, we downloaded 10 consecutive datasets (1999–2018) from the NHANES to precisely evaluate the association between RAR and all-cause mortality among stroke survivors. Initially, 101,316 individuals were included. The exclusion criteria are outlined below: under 20 years of age (n = 46,235); missing or unconfirmed stroke diagnosis (n = 52,884); missing RAR data (n = 359). Upon applying these criteria, 1838 individuals were incorporated into the final analysis (Fig. 1).

### 2.3. Measurement of RAR

During the NHANES survey, peripheral blood RDW (%) was measured using a Coulter analyzer at the Mobile Examination Center. ALB levels (g/dL) were assessed via the bromocresol purple method. The RAR was calculated according to the previously established formula: RAR = RDW (%)/ALB (g/dL).^[26]^ Participants were divided into 4 groups according to the quartiles of RAR: Q1: <2.98, Q2: 2.98–3.23, Q3: 3.23–3.58, and Q4: ≥3.58, with the RAR < 2.98 group serving as the reference group.

### 2.4. Stroke assessment

Stroke diagnosis was determined through self-reported responses obtained during in-person interviews, where participants were asked if a healthcare provider had ever confirmed a stroke diagnosis. Those who responded “yes” to the question, “Has a doctor or healthcare professional ever diagnosed you with a stroke?” were categorized as stroke survivors.^[27–29]^

### 2.5. Determination of mortality

Mortality data were obtained from the publicly accessible NHANES mortality files using exact serial number matching for each respondent. These files were derived from the National Death Index. If no match was identified in the National Death Index, the individual was assumed to be alive. The interval between the NHANES household interview date and the date of last verified vital status was computed as the event timeline for each participant. The follow-up period concluded on December 31, 2019. All-cause mortality was characterized as death resulting from any cause.

### 2.6. Covariates

This study incorporated covariates that may influence the association between RAR and all-cause mortality, including demographic factors: gender, age, race, education level, marital status, family income-to-poverty ratio (PIR), body mass index (BMI), drink, smoking status was categorized as current smokers (having smoked ≥ 100 cigarettes and currently smoking), former smokers (having smoked ≥ 100 cigarettes but quit), or never smokers (having smoked < 100 cigarettes or never smoked).^[30]^ Lifestyle factors encompass physical activity, calculated through metabolic equivalents (MET) scoring according to activity type and intensity. The MET value was multiplied by the average duration and frequency over the previous 30 days to calculate the MET of each activity per 30 days (MET min/30d). These MET min/30d values were aggregated and then divided by 4.29 to calculate the total weekly MET minutes. Before conducting the analysis, participants were grouped into low-intensity or high-intensity based on whether they met national physical activity standards (low-intensity < 500 MET/wk; high-intensity ≥ 500 MET/wk).^[31]^ Laboratory measures included ALB (g/dL), hemoglobin (g/dL), RDW (%), total cholesterol (mg/dL), and uric acid (mg/dL). Chronic disease history included: diabetes, defined as a history of diabetes, use of insulin, use of glucose-lowering medications, hemoglobin A1c ≥ 6.5%, fasting glucose ≥ 126 mg/dL, or 2-hour postprandial glucose ≥ 200 mg/dL (meeting any one criterion).^[32]^ Hypertension, defined as a history of hypertension, systolic blood pressure > 140 mm Hg, or diastolic blood pressure > 90 mm Hg (meeting any one criterion). Cardiovascular disease (CVD) included a history of congestive heart failure, coronary artery disease, angina, or heart attack.

### 2.7. Statistical analysis

Considering the intricate, multistage probability sampling design of NHANES for selecting representative participants, we integrated primary sampling units, sample weights, and stratification into the data analysis. Weighted analyses were conducted using the “survey” package in R software to derive nationally representative estimates. This method ensures that the study results are applicable to the noninstitutionalized United States population and avoids overestimating statistical significance. Adhering to NHANES guidelines, weight selection prioritized variables representing smaller population subgroups, with weights assigned accordingly. For missing data, linear and logistic regression techniques were applied for imputation, with nearest neighbor values derived based on the predictive mean matching. In this study, baseline characteristics were categorized into quartiles based on RAR. Continuous variables were reported as weighted means with standard error, and categorical variables were expressed as percentages. Analysis of variance and Chi-square tests were utilized to evaluate differences in baseline characteristics across groups. Multivariable Cox regression models were applied to examine the relationship between RAR and all-cause mortality risk, calculating hazard ratios (HRs) and 95% confidence intervals (CIs). The proportional hazards assumption was evaluated using the Schoenfeld residuals approach. Baseline variables with a *P*-value < .2 in univariate analysis or considered clinically relevant to outcomes were included in the multivariable Cox regression models. An unadjusted crude model (Model 1) was initially constructed. Model 2 was adjusted for gender, age, and race. Model 3 adjusted for all covariates on the basis of Model 2. Survival analysis was performed using the Kaplan–Meier approach, with between-group comparisons conducted via the log-rank test. Additionally, restricted cubic spline plots were employed to investigate potential nonlinear relationships. A two-stage linear regression model was utilized to confirm the nonlinear association between RAR and all-cause mortality for threshold effect analysis. To verify the consistency of the relationship between RAR and stroke risk across various subgroups, subgroup analyses and interaction tests were carried out. To assess the reliability of our results, sensitivity analyses were conducted by applying logarithmic transformation to RAR and using unweighted data. Statistical significance was defined at *P* < .05. Data processing and statistical analyses were executed using R software (version 4.4.0; R Foundation for Statistical Computing, Vienna, Austria) (April 24, 2024).

## 3. Results

### 3.1. Weighted baseline characteristics of the study population

As depicted in Table 1, the analysis included data from 1838 stroke survivors drawn from a cohort of 101,316 individuals. During a median follow-up duration of 6.42 years, 861 deaths were recorded. RAR was categorized into quartiles. The distribution across quartiles was as follows: Q1 included 371 individuals (20.18%), Q2 included 459 individuals (24.97%), Q3 included 496 individuals (26.99%), and Q4 included 512 individuals (27.86%). Notable statistical differences (*P* < .05) were identified across the 4 groups for age, ALB, hemoglobin, RDW, total cholesterol, RAR, gender, race, marital status, BMI, physical activity, CVD, all-cause mortality, hypertension, and diabetes. No statistically significant differences (*P* > .05) were found for uric acid, education level, PIR, smoking status, or drink. Comparative analysis revealed that 49.99% of participants in Q4 had a BMI ≥ 30 kg/m², significantly higher than 22.65% in Q1. The mean serum total cholesterol in Q4 was 181.24 mg/dL, lower than 196.30 mg/dL in Q1. The proportion of participants with a PIR ≥ 3.5 showed a declining trend from Q1 to Q4.

**Table 1:** Baseline characteristics of the participants.

Variable	Q1 (n = 371)	Q2 (n = 459)	Q3 (n = 496)	Q4 (n = 512)	*P*
RAR, mean (SE)	2.80 (0.01)	3.10 (0.00)	3.39 (0.01)	4.06 (0.03)	<.001
Age (yr), mean (SE)	59.55 (1.07)	64.47 (0.81)	67.16 (0.71)	67.99 (0.78)	<.001
**Gender, n (%**)					<.001
Male	214 (53.87)	221 (37.85)	245 (40.56)	231 (37.71)	
Female	157 (46.13)	238 (62.15)	251 (59.44)	281 (62.29)	
**Race/ethnicity, n (%**)					<.001
Mexican American	69 (5.33)	50 (4.24)	53 (4.66)	34 (2.80)	
Non-Hispanic White	221 (77.42)	257 (75.46)	246 (68.62)	238 (65.52)	
Non-Hispanic Black	36 (4.39)	97 (11.80)	143 (17.20)	186 (21.96)	
Other races	45 (12.86)	55 (8.50)	54 (9.53)	54 (9.73)	
**Education level, n (%**)					.245
<High school	134 (25.28)	164 (25.60)	189 (32.47)	207 (32.87)	
High school	93 (30.38)	122 (29.56)	120 (28.67)	134 (28.69)	
>High school	144 (44.34)	173 (44.84)	187 (38.86)	171 (38.44)	
**Marital status, n (%**)					.001
Married/living with partner	215 (65.25)	248 (53.48)	246 (52.23)	239 (53.18)	
Widowed/divorced/separated	125 (25.14)	178 (39.40)	211 (39.45)	230 (40.73)	
Never married	31 (9.61)	33 (7.13)	39 (8.32)	43 (6.09)	
**PIR, n (%**)					.274
<1.3	137 (29.47)	191 (35.28)	198 (33.01)	215 (34.04)	
≥ 1.3 < 3.5	153 (41.24)	194 (41.85)	212 (46.74)	204 (44.05)	
≥ 3.5	81 (29.29)	74 (22.87)	86 (20.25)	93 (21.91)	
**BMI, n (%**)					<.001
<25	107 (28.76)	107 (23.83)	117 (22.55)	119 (22.65)	
25–30	142 (37.51)	177 (39.89)	151 (27.94)	152 (27.36)	
≥30	122 (33.73)	175 (36.28)	228 (49.52)	241 (49.99)	
ALB, mean (SE)	4.45 (0.01)	4.22 (0.01)	4.04 (0.01)	3.76 (0.02)	<.001
UA, mean (SE)	5.65 (0.09)	5.66 (0.11)	5.86 (0.09)	5.90 (0.11)	.274
HGB, mean (SE)	14.53 (0.08)	14.21 (0.08)	13.86 (0.09)	12.78 (0.10)	<.001
RDW, mean (SE)	12.43 (0.03)	13.08 (0.03)	13.67 (0.04)	15.18 (0.10)	<.001
TC, mean (SE)	196.30 (2.83)	193.96 (2.72)	190.62 (2.95)	181.24 (2.62)	<.001
**Smoking status, n (%**)					.483
Nonsmoker	166 (43.96)	190 (39.03)	206 (45.87)	190 (39.29)	
Former smoker	130 (32.68)	164 (34.35)	177 (30.65)	203 (37.02)	
Current smoker	75 (23.36)	105 (26.62)	113 (23.49)	119 (23.69)	
**Drink, n (%**)					.727
No	132 (33.18)	162 (33.95)	186 (37.34)	182 (34.26)	
Yes	239 (66.82)	297 (66.05)	310 (62.66)	330 (65.74)	
**Physical activity, n (%**)					<.001
Low physical activity	183 (44.03)	265 (57.34)	324 (61.24)	356 (67.95)	
High physical activity	188 (55.97)	194 (42.66)	172 (38.76)	156 (32.05)	
**High blood pressure, n (%**)					<.001
No	85 (29.23)	94 (22.83)	94 (19.89)	75 (14.07)	
Yes	286 (70.77)	365 (77.17)	402 (80.11)	437 (85.93)	
**Diabetes, n (%**)					<.001
No	253 (73.64)	295 (68.69)	288 (60.91)	263 (52.43)	
Yes	118 (26.36)	164 (31.31)	208 (39.09)	249 (47.57)	
**CVD, n (%**)					.031
Yes	119 (31.63)	172 (36.74)	191 (37.13)	225 (44.43)	
No	252 (68.37)	287 (63.26)	305 (62.87)	287 (55.57)	
**All-cause death, n (%**)					.114
No	200 (61.62)	261 (60.67)	261 (54.82)	255 (54.06)	
Yes	171 (38.38)	198 (39.33)	235 (45.18)	257 (45.94)	

ALB = albumin, BMI = body mass index, CVD = cardiovascular disease, HGB = hemoglobin, PIR = family income-to-poverty ratio, RAR = red cell distribution width to albumin ratio, RDW = red cell distribution width, SE = standard error, TC = total cholesterol, UA = uric acid.

### 3.2. Association between RAR and all-cause mortality in stroke survivors

Univariate and multivariable Cox regression analyses indicated that elevated RAR levels were linked to increased all-cause mortality (see Table S1, Supplemental Digital Content, https://links.lww.com/MD/R111, which illustrates the results of Univariate and Multivariate Cox Proportional Hazards Models). RAR was evaluated as both a continuous and categorical variable (divided into quartiles). A significant positive relationship between RAR and all-cause mortality in stroke survivors was detected across Models 1, 2, and 3 (Table 2). Specifically, Model 1 produced an HR of 1.87 (95% CI: 1.64–2.13), and Model 2 produced an HR of 1.87 (95% CI: 1.61–2.16). In the fully adjusted multivariable model (Model 3), each 1-unit increase in RAR corresponded to a 1.67-fold higher risk of all-cause mortality among stroke survivors (HR = 1.67, 95% CI: 1.41–1.98). When we evaluated RAR as a categorical variable divided into quartiles, this association remained statistically significant (*P* for trend < .05 across all models). In Model 3, stroke survivors in the highest RAR quartile (Q4) exhibited a 1.95-fold higher risk of all-cause mortality compared with those in the lowest quartile (Q1) (HR = 1.95, 95% CI: 1.48–2.56).

**Table 2:** Associations between RAR and Pca all-cause mortality in stroke survivors.

Exposure	Non-adjusted model (Model 1)	Incomplete adjusted model (Model 2)	Fully adjusted model (Model 3)
	HR (95% CI) *P*-value	HR (95% CI) *P*-value	HR (95% CI) *P*-value
RAR	1.87 (1.64–2.13) <.001	1.87 (1.61–2.16) <.001	1.67 (1.41–1.98) <.001
RAR 4			
Q1	1.00 (Reference)	1.00 (Reference)	1.00 (Reference)
Q2	1.33 (1.05–1.69) .018	1.09 (0.87–1.37) .435	0.99 (0.79–1.24) .899
Q3	1.99 (1.54–2.56) <.001	1.64 (1.26–2.14) <.001	1.44 (1.10–1.89) .008
Q4	2.83 (2.20–3.63) <.001	2.33 (1.79–3.04) <.001	1.95 (1.48–2.56) <.001
*P* for trend	<.001	<.001	.001

Model 1: Crude; Model 2: adjusted for gender, race and age; Model 3: adjusted for gender, race, age, education level, marital status, PIR, BMI, physical activity, smoking status, drink, high blood pressure, diabetes, CVD, UA, HGB, and TC.

BMI = body mass index, CI = confidence interval, CVD = cardiovascular disease, HGB = hemoglobin, HR = hazard ratio, PIR = family income-to-poverty ratio, RAR = red cell distribution width to albumin ratio, TC = total cholesterol, UA = uric acid.

### 3.3. Nonlinear association between RAR and all-cause mortality in stroke survivors

**Figure 2.**
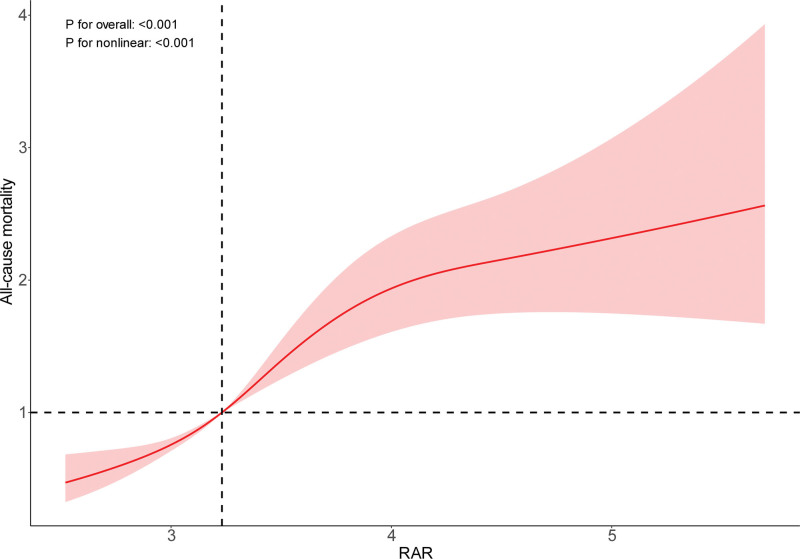
Dose–response relationship between RAR and all-cause mortality in stroke survivors (RCS analysis). RAR = red cell distribution width to albumin ratio, RCS = restricted cubic spline.

**Figure 3.**
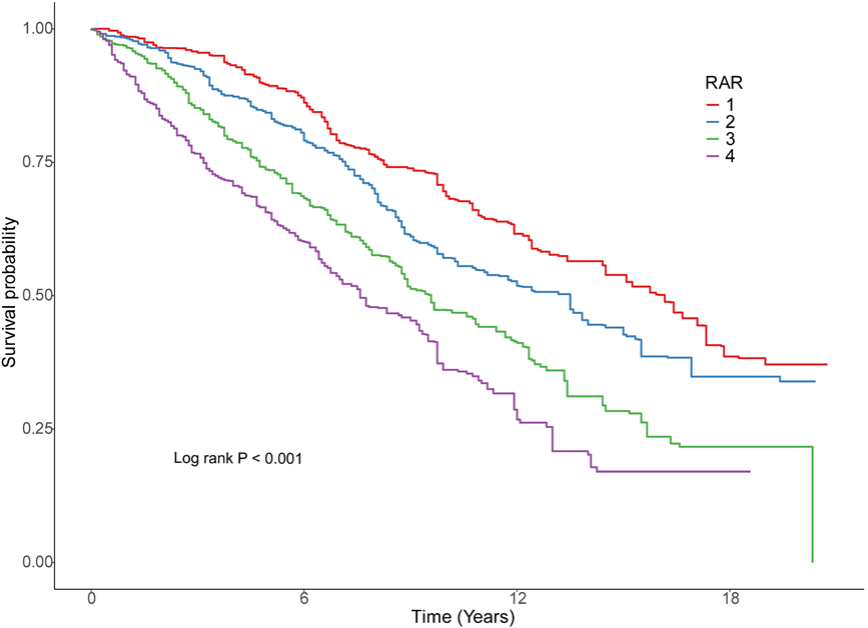
Kaplan–Meier curves for RAR and all-cause mortality in stroke survivors. RAR = red cell distribution width to albumin ratio.

We utilized restricted cubic spline analysis to investigate the nonlinear relationship between RAR and all-cause mortality. As depicted in Figure 2, the overall association was statistically significant (*P* < .001), with evidence of nonlinearity (*P* for nonlinearity = .001), confirming a nonlinear relationship between RAR and stroke outcomes. Threshold analysis conducted using the segmented package identified a threshold effect in the association between RAR and mortality (*P* for likelihood ratio test < .001) (Table 3). Overall, RAR was positively associated with mortality (HR = 1.77, 95% CI: 1.56–2.01). Specifically, when RAR was below 4.24, a significant positive association with all-cause mortality was observed (HR = 2.16, 95% CI: 1.77–2.64). However, when RAR exceeds 4.24, the association between RAR and mortality is not statistically significant. Analysis of Kaplan–Meier survival curves (Fig. 3) indicated that stroke survivors in the highest RAR quartile (Q4) exhibited markedly higher overall mortality rates in subsequent years compared to those in quartiles Q1 to Q3 (*P* < .001).

**Table 3:** Using a two-segment linear regression model to analyze the threshold effect of RAR on all-cause mortality in stroke survivors.

Outcome	HR (95% CI)	*P*
Fitting model by standard linear regression	1.77 (1.56–2.01)	<.001
Fitting model by two-piecewise linear regression		
Inflection point	4.473	
<4.24	2.16 (1.77–2.64)	<.001
≥4.24	1.27 (0.60–2.71)	.531
*P* for likelihood test		<.001

Covariates involved in this model was the same as adjust III model presented.

CI = confidence interval, HR = hazard ratio, RAR = red cell distribution width to albumin ratio.

### 3.4. Receiver operating characteristic curve analysis

**Figure 4.**
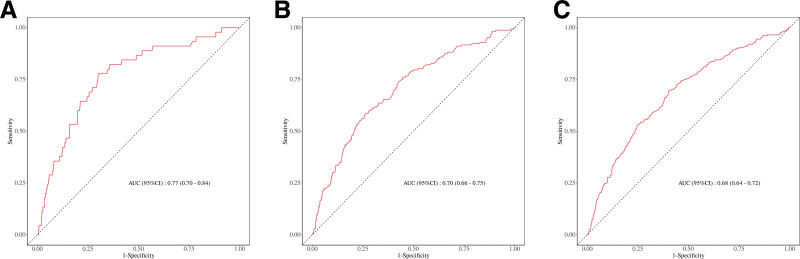
(A) The 1-year ROC curves of RAR for all-cause mortality in the training and validation groups. (B) The 3-year ROC curves of RAR for all-cause mortality in the training and validation groups. (C) The 5-year ROC curves of RAR for all-cause mortality in the training and validation groups. AUC = area under the curve, CI = confidence interval, RAR = red cell distribution width to albumin ratio, ROC = receiver operating characteristic.

The original stroke survivor dataset was randomly divided into a 7:3 ratio, with 1 subset allocated for model development (training set) and the other for model validation (validation set). Using a Cox regression prediction model, we generated receiver operating characteristic curves to evaluate the relationship between RAR and all-cause mortality in stroke survivors at 1, 3, and 5 years. As shown in Figure 4, the areas under the curve for both the training and validation sets at 1 year (A), 3 years (B), and 5 years (C) exceeded 0.6, indicating that RAR has moderate predictive value for all-cause mortality in stroke survivors.

### 3.5. Subgroup analysis

**Figure 5.**
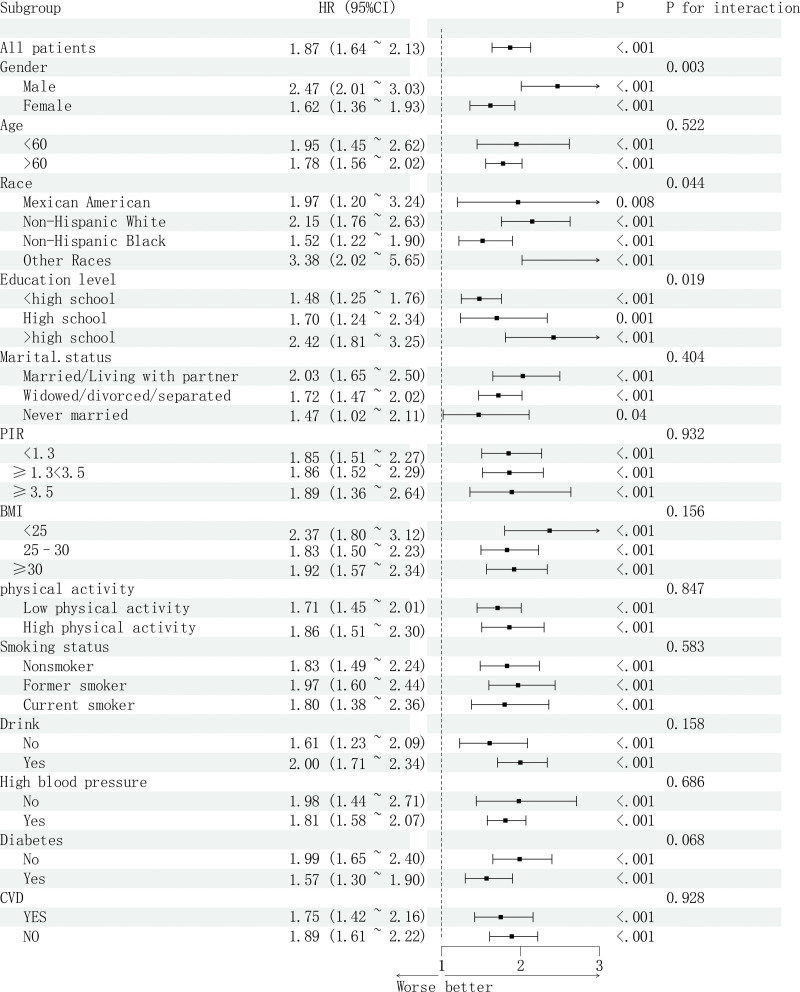
Forest plot of subgroup analysis of RAR and all-cause mortality in stroke survivors. BMI = body mass index, CI = confidence interval, CVD = cardiovascular disease, HR = hazard ratio, PIR = income-to-poverty ratio, RAR = red cell distribution width to albumin ratio.

We conducted subgroup analyses and examined the interactions between RAR and all-cause mortality in terms of gender, age, race, marital status, education level, family PIR, BMI, physical activity, hypertension, diabetes, CVD, drink, and smoking status (see Table S2, Supplemental Digital Content, https://links.lww.com/MD/R111, which illustrates the subgroup analysis of RAR and all-cause mortality in stroke survivors). As depicted in Figure 5, RAR was generally positively associated with all-cause mortality across all subgroups (HR = 1.87, 95% CI: 1.64–2.13). Notable interactions were identified across subgroups defined by gender and education level (*P* for interaction < .05). Among stroke survivors, as RAR levels increase, the increase in all-cause mortality is more significant in men than in women. The increase in all-cause mortality is more significant in other races than in Mexican Americans and non-Hispanic populations. Similarly, the growth rate of all-cause mortality with increasing RAR is significantly faster in people with an educational level higher than high school than in those with an educational level of high school or lower.

### 3.6. Sensitivity analysis

To evaluate the reliability of our results, we conducted sensitivity analyses by applying a logarithmic transformation to RAR values, followed by weighted logistic regression analysis (see Table S3, Supplemental Digital Content, https://links.lww.com/MD/R111, which illustrates the associations between logRAR and Pca all-cause mortality in stroke survivors). In Model 3, LogRAR, as a continuous variable, showed a positive correlation with all-cause mortality in stroke survivors (HR = 7.68, 95% CI: 3.91–15.09). Stroke survivors in the higher LogRAR quartiles exhibited increased risks of all-cause mortality (Q2: HR = 0.99, 95% CI: 0.79–1.24; Q3: HR = 1.44, 95% CI: 1.10–1.89; Q4: HR = 1.95, 95% CI: 1.48–2.56). Additionally, we conducted logistic regression analysis using unweighted data (see Table S4, Supplemental Digital Content, https://links.lww.com/MD/R111, which illustrates the associations between RAR and Pca all-cause mortality in stroke survivors [unweighted]). In Model 3, RAR as a continuous variable remained positively associated with all-cause mortality risk in stroke survivors (HR = 1.77, 95% CI: 1.56–2.01), with higher RAR quartiles indicating elevated mortality risks (Q2: HR = 1.10, 95% CI: 0.90–1.34; Q3: HR = 1.53, 95% CI: 1.24–1.88; Q4: HR = 2.37, 95% CI: 1.90–2.96). These sensitivity analyses confirm that our findings are robust and reliable, demonstrating a stable relationship between RAR and all-cause mortality among stroke survivors.

## 4. Discussion

This research examined the relationship between RAR and all-cause mortality in stroke survivors. Leveraging extensive data from NHANES, the baseline cohort included 1838 stroke survivors, with 861 mortality cases recorded during the follow-up period. We observed a strong positive relationship between mortality risk and increasing RAR levels when RAR was below 4.24. Furthermore, subgroup analyses revealed that the relationship between RAR and stroke outcomes may differ across specific populations. Thus, RAR, as a more comprehensive composite indicator, holds significant value for predicting all-cause mortality risk in stroke survivors.

Multiple studies have demonstrated, that increased RDW is closely linked to stroke incidence, clinical outcomes, and complications. Söderholm et al^[33]^ found that individuals in the highest RDW quartile exhibited a 31% greater stroke occurrence compared to those in the lowest quartile (HR = 1.31, 95% CI: 1.11–1.54), and this association remained significant independent of traditional cardiovascular risk factors, including hypertension and diabetes. Within the context of hemorrhagic stroke, Lorente et al^[34]^ conducted a study involving 117 patients with spontaneous intracerebral hemorrhage, revealing that early RDW elevation was independently associated with 30-day mortality. This observation indicates that RDW may function as a new biomarker for mortality prediction, offering substantial clinical value. For patients with ischemic stroke, Eren et al^[35]^ identified high RDW levels at admission independently predicted mortality in individuals with acute anterior circulation ischemic stroke receiving endovascular therapy. Moreover, higher RDW was linked to an increased likelihood of reperfusion failure. Additionally, a multicenter study by Wu et al^[36]^ demonstrated that each 1-unit increase in RDW was associated with a 28% higher risk of poststroke gastrointestinal bleeding (OR = 1.28, 95% CI: 1.11–1.48). Krongsut et al^[37]^ also reported that higher RDW levels were strongly linked to an elevated likelihood of pneumonia and 3-month mortality in patients undergoing thrombolytic therapy.

The core mechanism underlying this association lies in the synergistic damage induced by inflammation and oxidative stress, which interact through neuro-hematological pathways to form a vicious cycle. Following stroke, ischemia/reperfusion injury swiftly triggers the central nervous system’s immune response, with microglia and astrocytes secreting substantial amounts of pro-inflammatory cytokines, such as tumor necrosis factor-α and interleukin-6, markedly elevating the risk of ischemic stroke.^[38]^ Numerous studies have shown that inflammatory injury is crucial in secondary brain damage caused by intracerebral hemorrhage. Post-intracerebral hemorrhage activation of the innate immune system initiates a cascade of inflammatory responses, including microglial activation, which aggravates brain injury by promoting tissue damage, blood–brain barrier dysfunction, and neuronal cell death.^[39]^ At the same time, inflammation triggers the secretion of pro-inflammatory cytokines, including tumor necrosis factor-α and interleukin-6, through activation of the nuclear factor-kappa B pathway, which contributes to increased apoptosis of erythroid progenitor cells.^[40]^ Furthermore, activated caspases directly cleave the transcription factor GATA-binding protein 1, impairing nuclear extrusion in late-stage erythroblasts and disrupting hemoglobin synthesis.^[41]^ In addition, inflammation-induced overexpression of hepcidin leads to functional iron deficiency, interfering with erythrocyte maturation and promoting the premature release of immature reticulocytes into circulation. The heterogeneity in reticulocyte volume contributes directly to elevated RDW levels.^[42]^ Supporting this mechanism, Liu et al reported that individuals in the high-RDW group displayed markedly elevated reticulocyte counts compared to those in the low-RDW group.^[43]^ Oxidative stress, acting as an “amplifier” of inflammation, further exacerbates stroke pathology through multiple mechanisms. Reactive oxygen species directly damage the phospholipid bilayer of red blood cell membranes, triggering lipid peroxidation, reducing membrane fluidity, and increasing osmotic fragility. These alterations stimulate compensatory release of immature reticulocytes from the bone marrow, ultimately contributing to elevated RDW. Notably, red blood cell heterogeneity is closely correlated with the degree of oxidative damage.^[44]^ Additionally, reactive oxygen species activate matrix metalloproteinases, degrade basement membrane collagen, oxidize tight junction proteins in endothelial cells, and induce endothelial apoptosis (collectively compromising blood–brain barrier integrity and increasing the risk of post-reperfusion blood–brain barrier leakage).^[45,46]^ Systemically, oxidative stress damages the gastric mucosal barrier and impairs alveolar immune function, thereby promoting poststroke complications such as gastrointestinal bleeding and pneumonia.^[47,48]^ Elevated RDW, which reflects increased red cell heterogeneity, may further intensify oxidative damage by enhancing blood viscosity and releasing free heme, ultimately forming a cascade of “neuroinflammation–hematologic abnormalities–multiorgan complications.”

ALB, produced by the liver, acts as a critical indicator of nutritional status and provides neuroprotective benefits through various mechanisms, including anti-inflammatory, antioxidant, and vascular homeostasis regulation. Specifically, the cysteine-34 thiol group in its molecular structure directly scavenges reactive oxygen species and nitrogen species and chelates free metal ions (e.g., copper and iron) to inhibit the Fenton reaction, thereby blocking upstream pathways of oxidative stress.^[49,50]^ Additionally, sulfur-containing amino acids derived from ALB degradation provide precursors for glutathione synthesis, indirectly enhancing mitochondrial antioxidant capacity.^[51]^ ALB also regulates nitric oxide signaling and arachidonic acid metabolism to maintain vascular endothelial function and suppress excessive inflammation.^[52]^ Numerous studies have confirmed that ALB levels are an independent predictor of functional outcomes and all-cause mortality in stroke patients. For instance, Deng et al^[53]^ found that patients with low ALB levels are more likely to experience poor functional outcomes, with those having ALB ≤ 40 g/L facing a 1.48-fold higher risk of all-cause mortality compared to those with ALB > 40 g/L (95% CI: 1.19–1.84, *P* < .01). This study further revealed that ALB levels in the highest RAR quartile (3.89 ± 0.02 g/dL) were significantly lower than those in the lowest quartile (4.48 ± 0.02 g/dL). In conclusion, ALB acts as a key protective factor in stroke through nutritional support, antioxidant activity, and inflammation modulation, serving as an independent biomarker for assessing tissue damage, predicting prognosis, and guiding personalized clinical interventions.

The RAR integrates the dynamic balance between inflammatory damage (RDW) and protective mechanisms (ALB), with an elevated RAR indicating a decompensated state where “damage exceeds compensation.” In this study, a nonlinear relationship between RAR and all-cause mortality (threshold: 4.24) suggests that when RAR < 4.24, mortality risk is primarily driven linearly by inflammatory burden. However, when RAR ≥ 4.24, oxidative stress surpasses the threshold, triggering apoptosis and multi-organ failure, at which point ALB’s protective effects are completely offset.

The RAR, as a novel composite index combining RDW (inflammatory burden) and ALB (protective capacity), holds significant value in quantitatively assessing the dynamic balance of “inflammation-oxidation-nutrition” in the body. This research reveals a notable nonlinear positive relationship between RAR and all-cause mortality in stroke survivors. When RAR < 4.24, mortality risk increases linearly with the inflammatory burden. However, when RAR ≥ 4.24, oxidative stress exceeds the compensatory threshold, and mortality risk may be driven by multi-organ failure, at which point ALB’s protective effects are entirely offset.^[54–56]^ Subgroup analysis further reveals that RAR’s predictive performance is more pronounced in males and individuals with higher education levels, potentially due to sex-related physiological differences (e.g., estrogen’s anti-inflammatory effects) and variations in health management behaviors.^[57]^ This population heterogeneity suggests that clinical application of RAR should incorporate individual characteristics for risk stratification. Moreover, dynamic monitoring of RAR fluctuations (e.g., changes in the ratio at different time points poststroke) may more accurately capture the trajectory of disease progression, providing a more sensitive early warning signal for timely intervention.

As a derivative parameter of routine laboratory indices, the RAR provides cost-effective and readily accessible benefits, ideal for early risk stratification of stroke patients in primary healthcare settings. For instance, patients with RAR ≥ 3.47 can be identified as a “high inflammation-low protection” group, warranting early initiation of anti-inflammatory therapies (e.g., statins), nutritional support (e.g., enteral nutrition to maintain ALB ≥ 35 g/L), and complication prevention strategies (e.g., proton pump inhibitors to reduce gastrointestinal bleeding risk). Dynamic monitoring of RAR changes (e.g., weekly assessments poststroke) can also evaluate treatment response, guiding adjustments to the intensity of individualized interventions.

However, this study has certain limitations. First, the NHANES database’s cross-sectional design limits causal inference, and the relationship between RAR and mortality may be affected by unmeasured confounders. (e.g., genetic predisposition or long-term medication use). Second, the stroke diagnosis relied on self-reporting without imaging confirmation, potentially introducing case classification bias. Third, in this study, “all-cause mortality” was defined as the outcome measure, which included deaths resulting from all causes for analysis. While this approach offers advantages in terms of objectivity and completeness in data collection, its inclusion scope encompasses deaths unrelated to the hypothetical pathological mechanism of the study (e.g., accidents). This inevitably introduces the issue of “association dilution,” which may weaken the true association strength between the core variables and the outcome measure in the study, thereby posing a potential impact on the accurate interpretation of the research results. In addition, the study cohort was predominantly United States-based, and the applicability of the results to other ethnic groups, particularly Asian populations with inherently lower baseline ALB levels, requires cautious validation.

## 5. Conclusion

In summary, the RAR demonstrates a nonlinear association with all-cause mortality in stroke survivors. When RAR is below 4.24, mortality risk exhibits a significant positive correlation with increasing RAR. RAR shows potential as a biomarker for assessing mortality risk in stroke survivors, offering potential value for clinical risk stratification and the formulation of targeted interventions. Further prospective studies are needed to confirm these findings and investigate the underlying mechanisms.

## Author contributions

**Conceptualization:** Shaoru Xing, Yujia Huo, Yu Wang.

**Data curation:** Lin Zhang, Zhe Yang, Shaoru Xing.

**Project administration:** Yujia Huo, Yu Wang.

**Resources:** Hongxia Du.

**Software:** Lin Zhang, Zhe Yang.

**Supervision:** Hongxia Du.

**Validation:** Lin Zhang, Zhe Yang.

**Visualization:** Lin Zhang, Zhe Yang.

**Writing – original draft:** Lin Zhang, Zhe Yang.

**Writing – review & editing:** Hongxia Du.

## Supplementary Material


