# Supplementary material for: Association between red cell distribution width to albumin ratio and all-cause mortality in stroke survivors: An observational study
Source: Medicine (Baltimore). 2026 Jan 9;105(2):e47040. doi: 10.1097/MD.0000000000047040 (PMC12795008; doi:10.1097/MD.0000000000047040)
Supplement: Supplementary file 1 [file medi-105-e47040-s001.pdf]

Table S1 Results of Univariate and Multivariate Cox Proportional Hazards Models.

Variables	Single factor					Multiple factors				
	β	S.E	Z	*P*	HR (95%CI)	β	S.E	Z	*P*	HR (95%CI)
Age	0.07	0.00	14.90	<.001	1.08 (1.07 - 1.09)	0.07	0.01	11.45	<.001	1.08 (1.06 - 1.09)
Gender										
Male					1.00(Reference)					1.00(Reference)
Female	-0.25	0.09	-2.82	.005	0.78 (0.65 - 0.93)	-0.42	0.09	-4.64	<.001	0.66 (0.55 - 0.79)
Race/ethnicity										
Mexican American					1.00(Reference)					1.00 (Reference)
Non-Hispanic White	0.41	0.17	2.41	.016	1.50 (1.08 - 2.10)	0.13	0.17	0.75	.452	1.14 (0.81 - 1.60)
Non-Hispanic Black	0.01	0.19	0.05	.959	1.01 (0.70 - 1.46)	-0.22	0.20	-1.12	.265	0.80 (0.54 - 1.18)
Other Races	-0.15	0.26	-0.58	.562	0.86 (0.51 - 1.44)	-0.12	0.24	-0.51	.610	0.89 (0.56 - 1.41)
Education level										
<high school					1.00 (Reference)					1.00 (Reference)
High school	-0.18	0.12	-1.54	.123	0.84 (0.67 - 1.05)	0.00	0.11	0.02	.983	1.00 (0.81 - 1.24)
>high school	-0.44	0.11	-3.89	<.001	0.64 (0.52 - 0.80)	-0.13	0.11	-1.22	.223	0.88 (0.71 - 1.08)
Marital.status										
Married/Living with partner					1.00(Reference)					1.00(Reference)
Widowed/ divorced/ separated	0.51	0.09	5.47	<.001	1.66 (1.39 - 1.99)	0.14	0.09	1.51	.131	1.15 (0.96 - 1.39)
Never married	-0.36	0.21	-1.70	.089	0.70 (0.46 - 1.06)	0.35	0.25	1.41	.160	1.41 (0.87 - 2.29)
PIR										

<1.3					1.00 (Reference)					1.00 (Reference)
≥ 1.3<3.5	0.30	0.12	2.57	.010	1.35 (1.07 - 1.69)	0.18	0.13	1.42	.154	1.20 (0.93 - 1.54)
≥ 3.5	-0.39	0.13	-2.93	.003	0.68 (0.52 - 0.88)	-0.10	0.14	-0.71	.477	0.91 (0.69 - 1.19)
Smoking status										
Nonsmoker					1.00 (Reference)					1.00(Reference)
Former smoker	0.28	0.10	2.74	.006	1.33 (1.08 - 1.63)	0.13	0.11	1.17	.242	1.14 (0.91 - 1.42)
Current smoker	-0.30	0.13	-2.29	.022	0.74 (0.57 - 0.96)	0.54	0.14	3.72	<.001	1.71 (1.29 - 2.27)
Drink										
No					1.00 (Reference)					1.00 (Reference)
Yes	-0.24	0.10	-2.46	.014	0.79 (0.65 - 0.95)	0.01	0.10	0.06	.951	1.01 (0.82 - 1.23)
BMI										
<25					1.00(Reference)					1.00(Reference)
25–30	-0.05	0.11	-0.45	.656	0.95 (0.76 - 1.18)	-0.16	0.11	-1.47	.141	0.86 (0.70 - 1.05)
≥30	-0.38	0.12	-3.18	.001	0.68 (0.54 - 0.86)	-0.34	0.12	-2.90	.004	0.71 (0.56 - 0.89)
physical activity, n(%)										
Low physical activity					1.00(Reference)					1.00 (Reference)
High physical activity	-0.77	0.10	-7.65	<.001	0.46 (0.38 - 0.57)	-0.48	0.10	-4.72	<.001	0.62 (0.51 - 0.76)
CVD										
Yes					1.00(Reference)					1.00 (Reference)
No	-0.49	0.09	-5.56	<.001	0.61 (0.51 - 0.73)	-0.37	0.08	-4.57	<.001	0.69 (0.59 - 0.81)
High blood pressure										
No					1.00 (Reference)					1.00(Reference)
Yes	0.44	0.12	3.77	<.001	1.55 (1.24 - 1.95)	-0.22	0.11	-2.02	.044	0.80 (0.65 - 0.99)

Diabetes										
No					1.00(Reference)					1.00(Reference)
Yes	0.34	0.10	3.58	<.001	1.41 (1.17 - 1.70)	0.16	0.11	1.48	.140	1.18 (0.95 - 1.46)
UA	0.14	0.03	5.52	<.001	1.15 (1.09 - 1.21)	0.04	0.02	1.80	.072	1.04 (1.00 - 1.09)
HGB	-0.16	0.03	-5.32	<.001	0.85 (0.81 - 0.91)	-0.05	0.04	-1.45	.147	0.95 (0.88 - 1.02)
TC	-0.01	0.00	-2.07	.038	0.99 (0.99 - 0.99)	0.00	0.00	1.66	.096	1.00 (1.00 - 1.00)
RAR	0.62	0.07	9.40	<.001	1.87 (1.64 - 2.13)	0.51	0.09	5.90	<.001	1.67 (1.41 - 1.98)

BMI=body mass index,CI=confidence interval CVD=cardiovascular disease,HGB=hemoglobin,HR=hazard ratio,PIR=family income-to-poverty ratio,RAR=red cell distribution width to albumin ratio,SE=standard error,TC=total cholesterol,UA=uric acid.

Table S2 Subgroup Analysis of RAR and All-Cause Mortality in Stroke Survivors.

Variables	HR (95%CI)	P	P for interaction
All patients	1.87 (1.64 ~ 2.13)	<.001	
Gender			.003
Male	2.47 (2.01 ~ 3.03)	<.001	
Female	1.62 (1.36 ~ 1.93)	<.001	
Age			.522
<60	1.95 (1.45 ~ 2.62)	<.001	
>60	1.78 (1.56 ~ 2.02)	<.001	
Race			.044
Mexican American	1.97 (1.20 ~ 3.24)	.008	
Non-Hispanic White	2.15 (1.76 ~ 2.63)	<.001	
Non-Hispanic Black	1.52 (1.22 ~ 1.90)	<.001	
Other Races	3.38 (2.02 ~ 5.65)	<.001	
Education level			.019
<high school	1.48 (1.25 ~ 1.76)	<.001	
High school	1.70 (1.24 ~ 2.34)	.001	
>high school	2.42 (1.81 ~ 3.25)	<.001	
Marital.status			.404
Married/Living with partner	2.03 (1.65 ~ 2.50)	<.001	
Widowed/ divorced/ separated	1.72 (1.47 ~ 2.02)	<.001	
Never married	1.47 (1.02 ~ 2.11)	.040	
PIR			.932
<1.3	1.85 (1.51 ~ 2.27)	<.001	
≥ 1.3<3.5	1.86 (1.52 ~ 2.29)	<.001	
≥ 3.5	1.89 (1.36 ~ 2.64)	<.001	

Smoking status			.583
Nonsmoker	1.83 (1.49 ~ 2.24)	<.001	
Former smoker	1.97 (1.60 ~ 2.44)	<.001	
Current smoker	1.80 (1.38 ~ 2.36)	<.001	
Drink			.158
No	1.61 (1.23 ~ 2.09)	<.001	
Yes	2.00 (1.71 ~ 2.34)	<.001	
BMI			.156
<25	2.37 (1.80 ~ 3.12)	<.001	
25–30	1.83 (1.50 ~ 2.23)	<.001	
≥30	1.92 (1.57 ~ 2.34)	<.001	
physical activity			.847
Low physical activity	1.71 (1.45 ~ 2.01)	<.001	
High physical activity	1.86 (1.51 ~ 2.30)	<.001	
CVD			.928
Yes	1.75 (1.42 ~ 2.16)	<.001	
No	1.89 (1.61 ~ 2.22)	<.001	
High blood pressure			.686
No	1.98 (1.44 ~ 2.71)	<.001	
Yes	1.81 (1.58 ~ 2.07)	<.001	
Diabetes			.068
No	1.99 (1.65 ~ 2.40)	<.001	
Yes	1.57 (1.30 ~ 1.90)	<.001	

BMI=body mass index,CI=confidence interval,CVD=cardiovascular disease, HR=hazard ratio,PIR=family income-to-poverty ratio,RAR=red cell distribution width to albumin ratio.

Table S3 Associations between logRAR and Pca All-cause mortality in stroke survivors.

Exposure	Non-adjusted model (model 1) HR(95%CI)P-value	Incomplete adjusted model (model 2) HR (95%CI) P-value	Fully adjusted model (model 3) HR (95%CI) P-value
logRAR	13.07 (7.88 - 21.66) <.001	12.07 (6.80 - 21.43) <.001	7.68 (3.91 - 15.09) .018
logRAR four			
Q1	1.00 (Reference)	1.00 (Reference)	1.00 (Reference)
Q2	1.33 (1.05 - 1.69) .018	1.09 (0.87 - 1.37) .435	0.99 (0.79 - 1.24) .899
Q3	1.99 (1.54 - 2.56) <.001	1.64 (1.26 - 2.14) <.001	1.44 (1.10 - 1.89) .008
Q4	2.83 (2.20 - 3.63) <.001	2.33 (1.79 - 3.04) <.001	1.95 (1.48 - 2.56) <.001
p for trend	<.001	<.001	.001

Model1: Crude;Model2: adjusted for gender, race and age; Model3: adjusted for gender, race, age, education level, marital status, PIR,BMI, physical activity, smoking status, drink, high blood pressure, diabetes, CVD, UA, HGB,TC.

BMI=body mass index,CI=confidence interval
CVD=cardiovascular disease,HGB=hemoglobin
HR=hazard ratio,PIR=family income-to-poverty ratio
RAR=red cell distribution width to albumin ratio, TC=total cholesterol,
UA=uric acid.

Table S4 Associations between RAR and Pca All-cause mortality in stroke survivors(Unweighted).

Exposure	Non-adjusted model(model 1) HR(95%CI)P-value	Incomplete adjusted model(model 2) HR (95%CI) P-value	Fully adjusted model(model 3) HR (95%CI) P-value
RAR	1.73 (1.57 ~ 1.91) <.001	1.89 (1.71 ~ 2.10) <.001	1.77 (1.56 ~ 2.01) <.001
RAR four			
Q1	1.00 (Reference)	1.00 (Reference)	1.00 (Reference)
Q2	1.30 (1.07 ~ 1.58) .008	1.22 (1.01 ~ 1.48) .045	1.10 (0.90 ~ 1.34) .341
Q3	1.59 (1.31 ~ 1.92) <.001	1.77 (1.45 ~ 2.16) <.001	1.53 (1.24 ~ 1.88) <.001
Q4	2.75 (2.27 ~ 3.33) <.001	2.90 (2.37 ~ 3.55) <.001	2.37 (1.90 ~ 2.96) <.001
p for trend	<.001	<.001	<.001

Model1: Crude;Model2: adjusted for gender, race and age; Model3: adjusted for gender, race, age, education level, marital status, PIR, BMI, physical activity, smoking status, drink, high blood pressure, diabetes, CVD, UA, HGB, TC.

BMI=body mass index,CI=confidence interval

CVD=cardiovascular disease,HGB=hemoglobin

HR=hazard ratio,PIR=family income-to-poverty ratio

RAR=red cell distribution width to albumin ratio, TC=total cholesterol,

UA=uric acid.
